# Supplementary material for: Live Triple Gene-Deleted Pseudorabies Virus-Vectored Subunit PCV2b and CSFV Vaccine Undergoes an Abortive Replication Cycle in the TG Neurons following Latency Reactivation
Source: Viruses. 2023 Feb 8;15(2):473. doi: 10.3390/v15020473 (PMC9963255; doi:10.3390/v15020473)
Supplement: Supplementary file 1 [file viruses-15-00473-s001.zip › Supplementary data PRVtmv+ latency reactivation study 12.07.2022.pptx]

## Slide 1
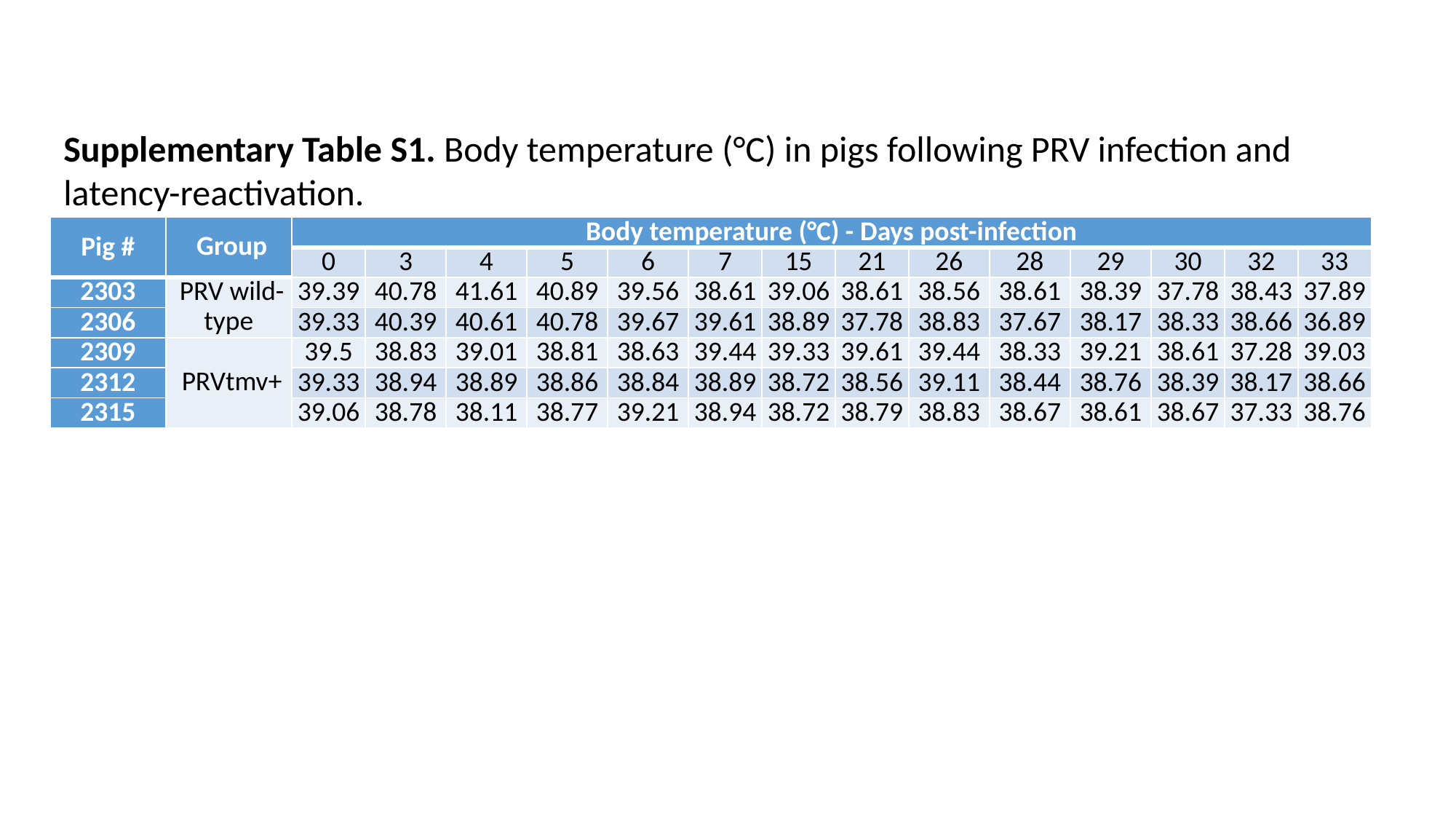

Supplementary Table S1. Body temperature (°C) in pigs following PRV infection and latency-reactivation.
| Pig # | Group | Body temperature (°C) - Days post-infection | | | | | | | | | | | | | |
| --- | --- | --- | --- | --- | --- | --- | --- | --- | --- | --- | --- | --- | --- | --- | --- |
| | | 0 | 3 | 4 | 5 | 6 | 7 | 15 | 21 | 26 | 28 | 29 | 30 | 32 | 33 |
| 2303 | PRV wild-type | 39.39 | 40.78 | 41.61 | 40.89 | 39.56 | 38.61 | 39.06 | 38.61 | 38.56 | 38.61 | 38.39 | 37.78 | 38.43 | 37.89 |
| 2306 | | 39.33 | 40.39 | 40.61 | 40.78 | 39.67 | 39.61 | 38.89 | 37.78 | 38.83 | 37.67 | 38.17 | 38.33 | 38.66 | 36.89 |
| 2309 | PRVtmv+ | 39.5 | 38.83 | 39.01 | 38.81 | 38.63 | 39.44 | 39.33 | 39.61 | 39.44 | 38.33 | 39.21 | 38.61 | 37.28 | 39.03 |
| 2312 | | 39.33 | 38.94 | 38.89 | 38.86 | 38.84 | 38.89 | 38.72 | 38.56 | 39.11 | 38.44 | 38.76 | 38.39 | 38.17 | 38.66 |
| 2315 | | 39.06 | 38.78 | 38.11 | 38.77 | 39.21 | 38.94 | 38.72 | 38.79 | 38.83 | 38.67 | 38.61 | 38.67 | 37.33 | 38.76 |

## Slide 2
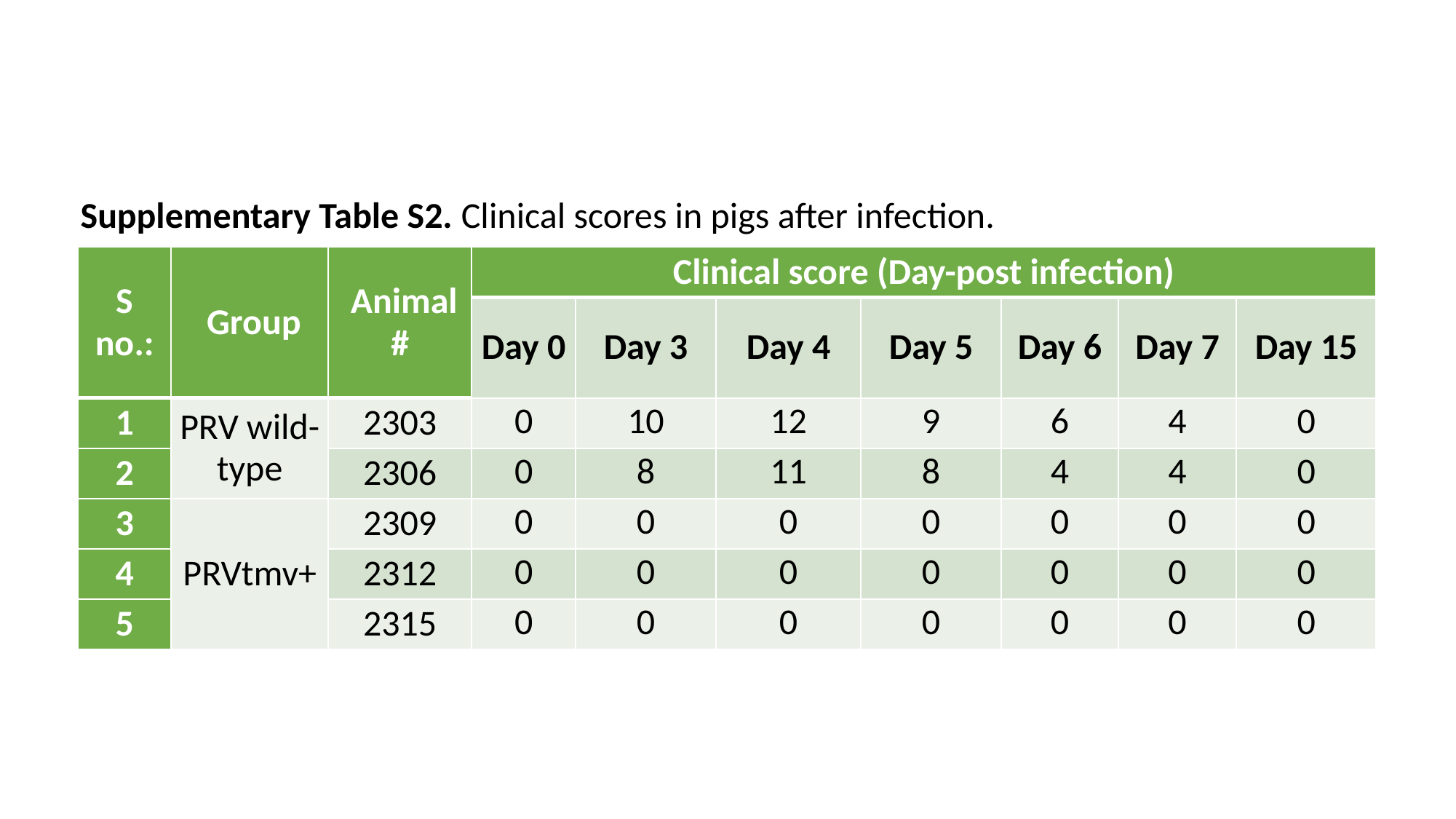

Supplementary Table S2. Clinical scores in pigs after infection.
| S no.: | Group | Animal # | Clinical score (Day-post infection) | | | | | | |
| --- | --- | --- | --- | --- | --- | --- | --- | --- | --- |
| | | | Day 0 | Day 3 | Day 4 | Day 5 | Day 6 | Day 7 | Day 15 |
| 1 | PRV wild-type | 2303 | 0 | 10 | 12 | 9 | 6 | 4 | 0 |
| 2 | | 2306 | 0 | 8 | 11 | 8 | 4 | 4 | 0 |
| 3 | PRVtmv+ | 2309 | 0 | 0 | 0 | 0 | 0 | 0 | 0 |
| 4 | | 2312 | 0 | 0 | 0 | 0 | 0 | 0 | 0 |
| 5 | | 2315 | 0 | 0 | 0 | 0 | 0 | 0 | 0 |

## Slide 3
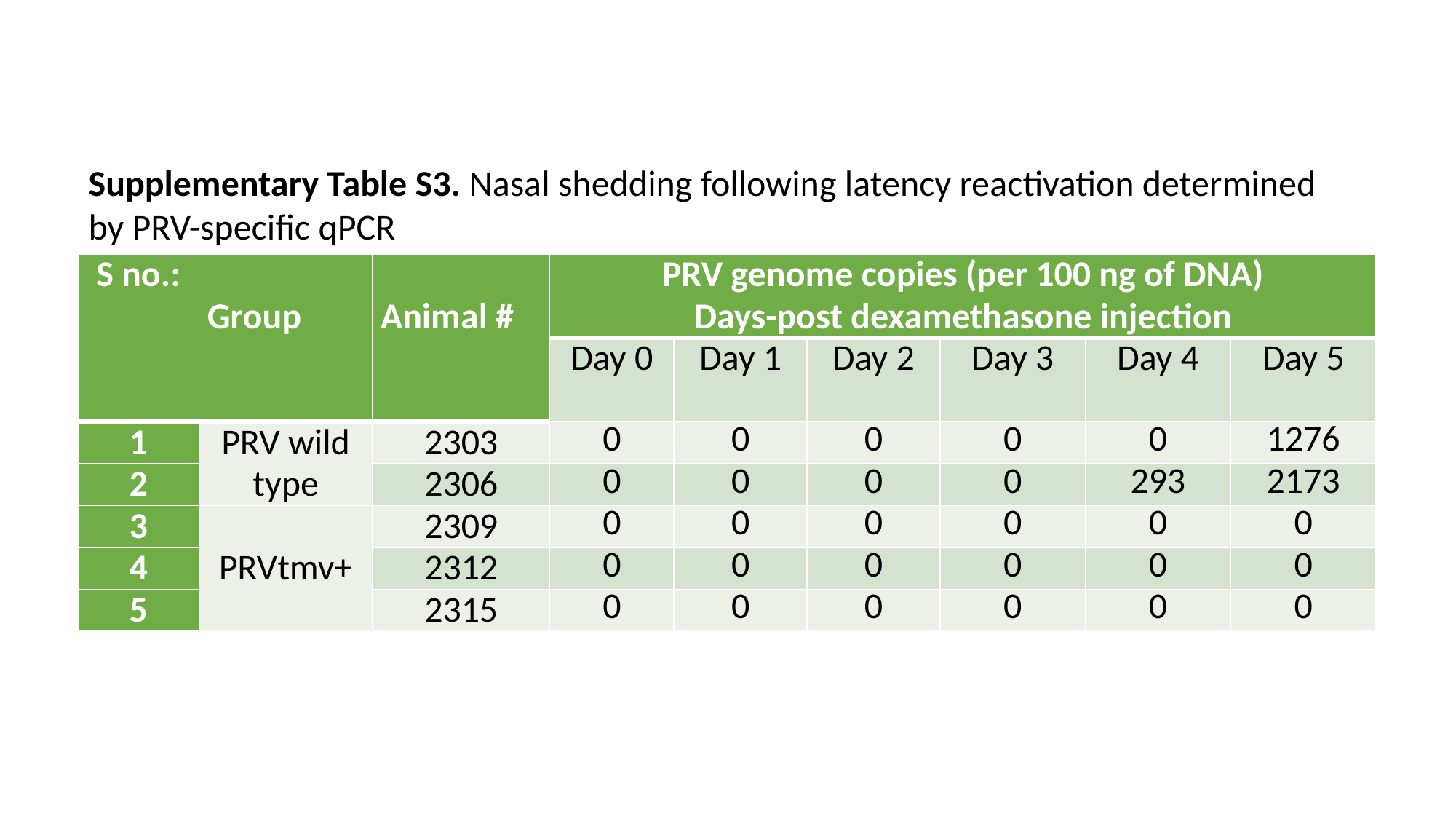

Supplementary Table S3. Nasal shedding following latency reactivation determined by PRV-specific qPCR
| S no.: | Group | Animal # | PRV genome copies (per 100 ng of DNA) Days-post dexamethasone injection | | | | | |
| --- | --- | --- | --- | --- | --- | --- | --- | --- |
| | | | Day 0 | Day 1 | Day 2 | Day 3 | Day 4 | Day 5 |
| 1 | PRV wild type | 2303 | 0 | 0 | 0 | 0 | 0 | 1276 |
| 2 | | 2306 | 0 | 0 | 0 | 0 | 293 | 2173 |
| 3 | PRVtmv+ | 2309 | 0 | 0 | 0 | 0 | 0 | 0 |
| 4 | | 2312 | 0 | 0 | 0 | 0 | 0 | 0 |
| 5 | | 2315 | 0 | 0 | 0 | 0 | 0 | 0 |

## Slide 4
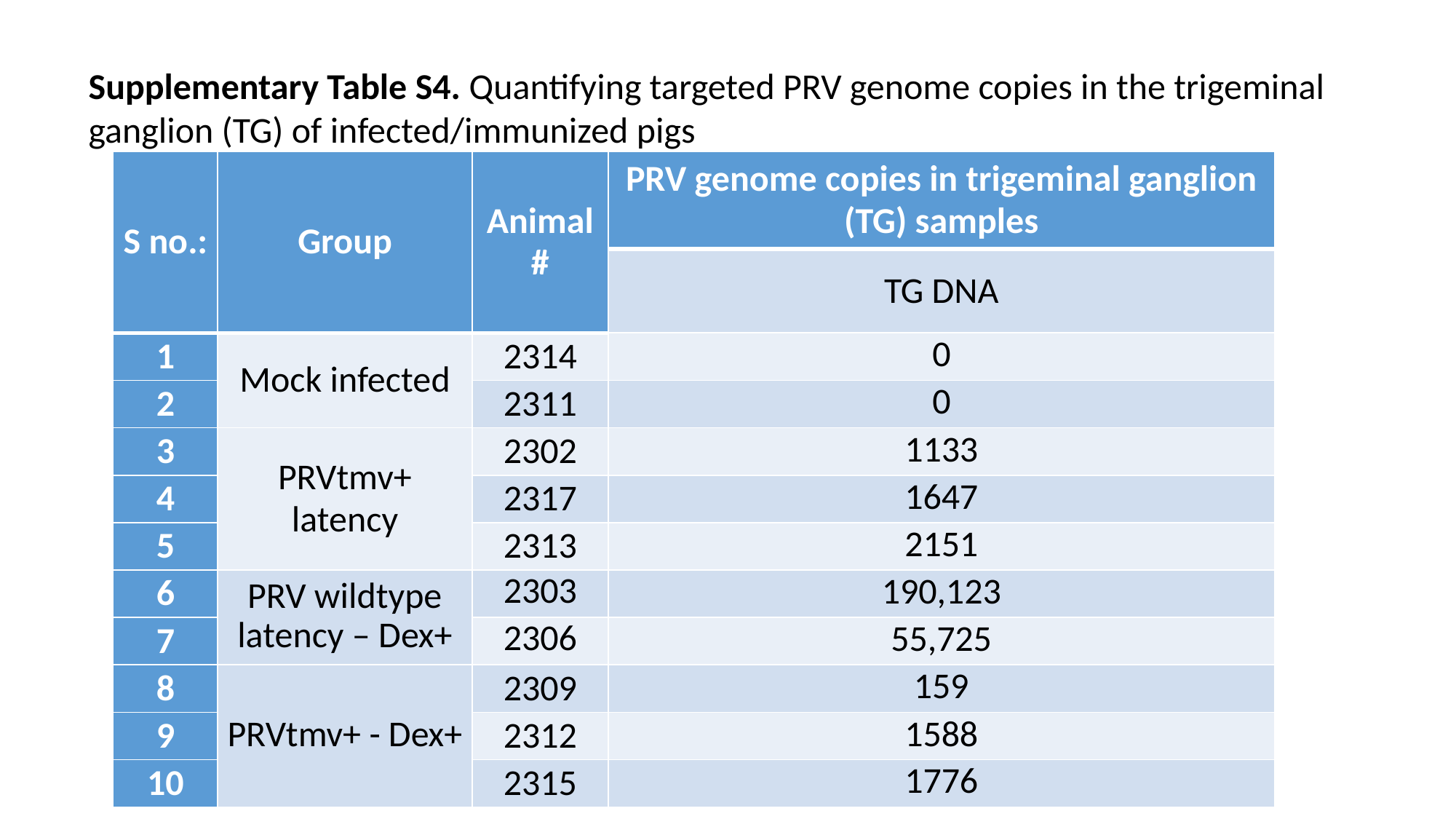

Supplementary Table S4. Quantifying targeted PRV genome copies in the trigeminal ganglion (TG) of infected/immunized pigs
| S no.: | Group | Animal # | PRV genome copies in trigeminal ganglion (TG) samples |
| --- | --- | --- | --- |
| | | | TG DNA |
| 1 | Mock infected | 2314 | 0 |
| 2 | | 2311 | 0 |
| 3 | PRVtmv+ latency | 2302 | 1133 |
| 4 | | 2317 | 1647 |
| 5 | | 2313 | 2151 |
| 6 | PRV wildtype latency – Dex+ | 2303 | 190,123 |
| 7 | | 2306 | 55,725 |
| 8 | PRVtmv+ - Dex+ | 2309 | 159 |
| 9 | | 2312 | 1588 |
| 10 | | 2315 | 1776 |

## Slide 5
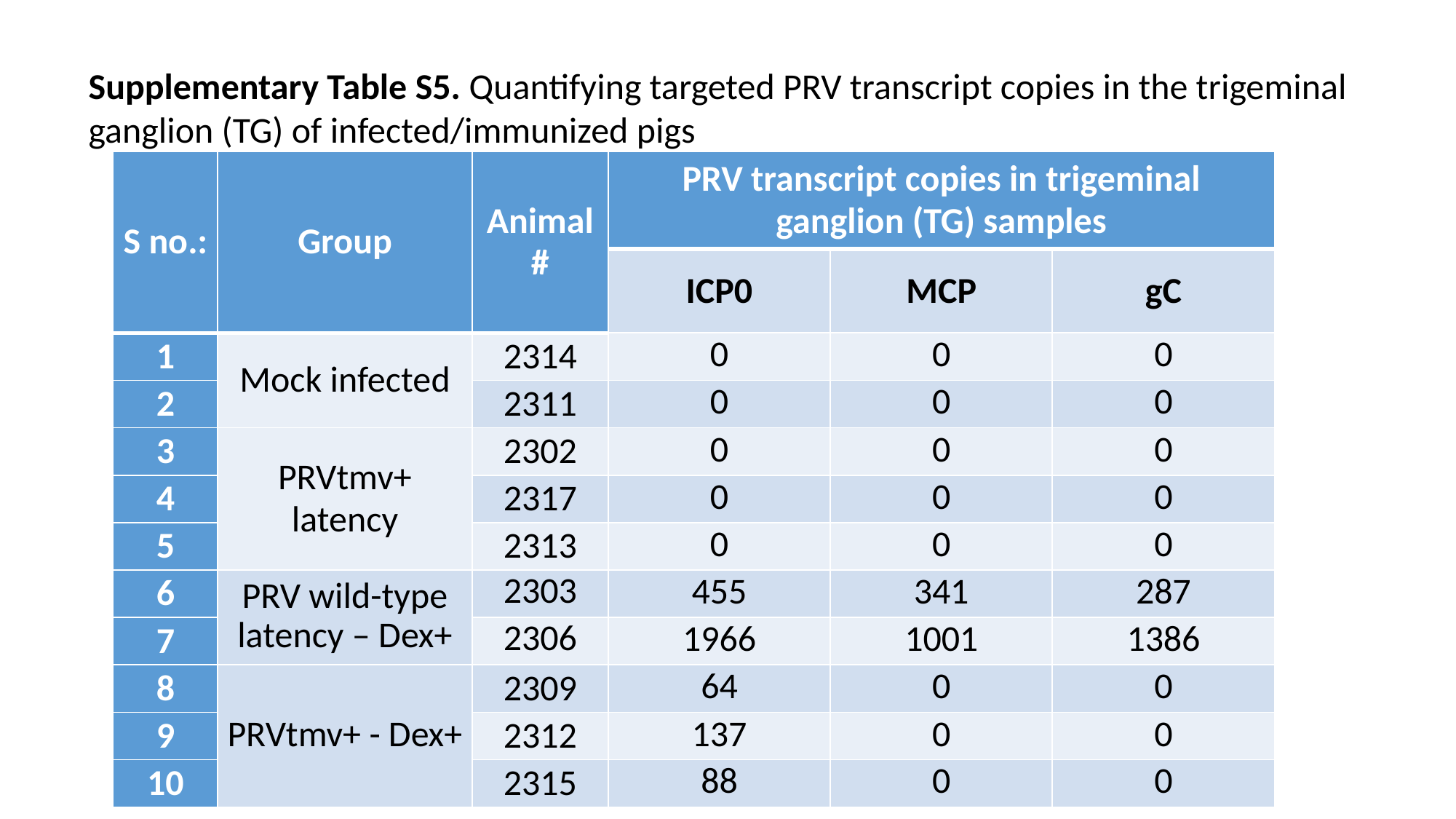

Supplementary Table S5. Quantifying targeted PRV transcript copies in the trigeminal ganglion (TG) of infected/immunized pigs
| S no.: | Group | Animal # | PRV transcript copies in trigeminal ganglion (TG) samples | | |
| --- | --- | --- | --- | --- | --- |
| | | | ICP0 | MCP | gC |
| 1 | Mock infected | 2314 | 0 | 0 | 0 |
| 2 | | 2311 | 0 | 0 | 0 |
| 3 | PRVtmv+ latency | 2302 | 0 | 0 | 0 |
| 4 | | 2317 | 0 | 0 | 0 |
| 5 | | 2313 | 0 | 0 | 0 |
| 6 | PRV wild-type latency – Dex+ | 2303 | 455 | 341 | 287 |
| 7 | | 2306 | 1966 | 1001 | 1386 |
| 8 | PRVtmv+ - Dex+ | 2309 | 64 | 0 | 0 |
| 9 | | 2312 | 137 | 0 | 0 |
| 10 | | 2315 | 88 | 0 | 0 |

## Slide 6
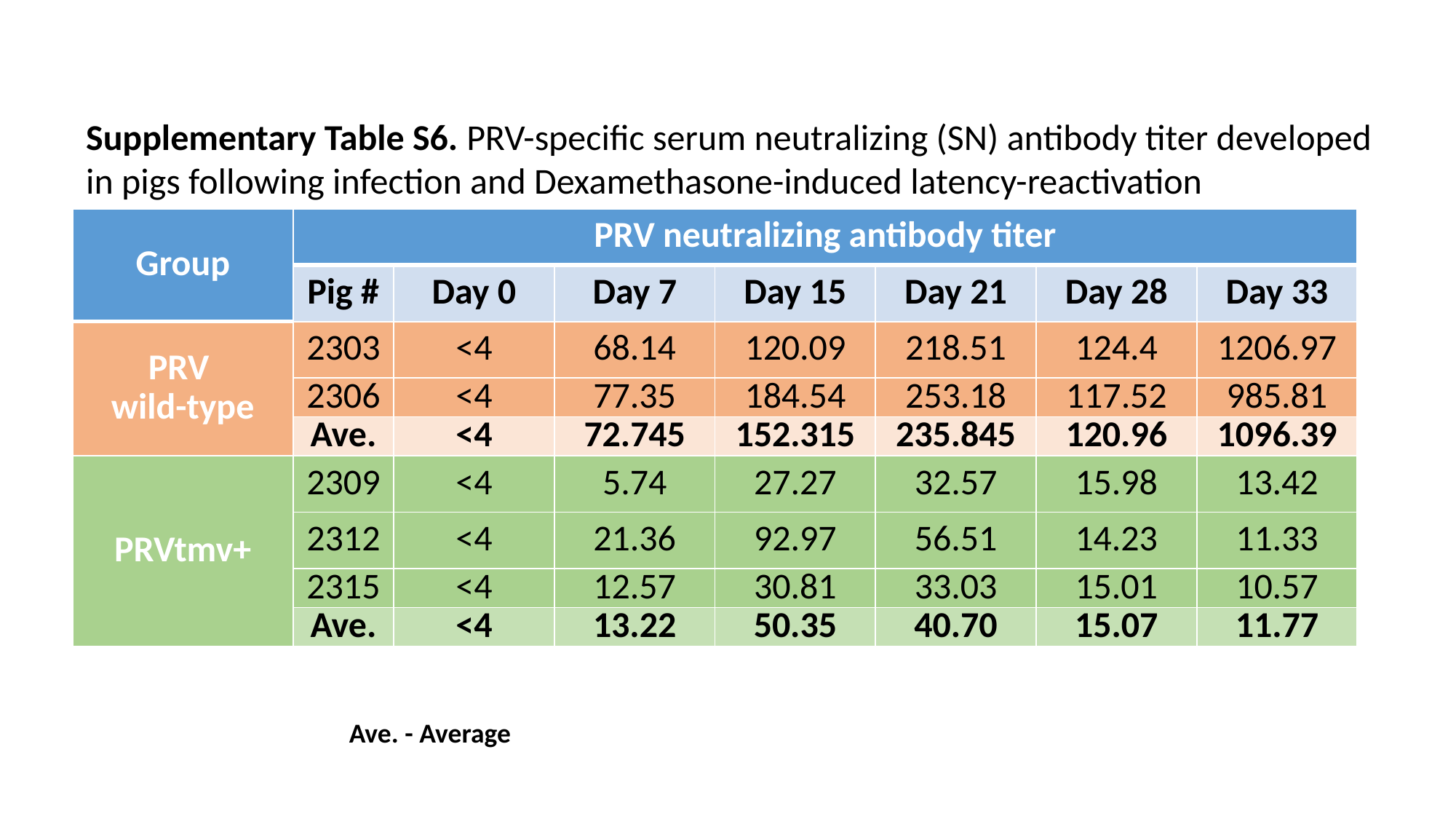

Supplementary Table S6. PRV-specific serum neutralizing (SN) antibody titer developed in pigs following infection and Dexamethasone-induced latency-reactivation
| Group | PRV neutralizing antibody titer | | | | | | |
| --- | --- | --- | --- | --- | --- | --- | --- |
| | Pig # | Day 0 | Day 7 | Day 15 | Day 21 | Day 28 | Day 33 |
| PRV wild-type | 2303 | ˂4 | 68.14 | 120.09 | 218.51 | 124.4 | 1206.97 |
| | 2306 | ˂4 | 77.35 | 184.54 | 253.18 | 117.52 | 985.81 |
| | Ave. | ˂4 | 72.745 | 152.315 | 235.845 | 120.96 | 1096.39 |
| PRVtmv+ | 2309 | ˂4 | 5.74 | 27.27 | 32.57 | 15.98 | 13.42 |
| | 2312 | ˂4 | 21.36 | 92.97 | 56.51 | 14.23 | 11.33 |
| | 2315 | ˂4 | 12.57 | 30.81 | 33.03 | 15.01 | 10.57 |
| | Ave. | ˂4 | 13.22 | 50.35 | 40.70 | 15.07 | 11.77 |
Ave. - Average
